# Scale-up of Direct-Acting Antiviral Treatment in Prisons Is Both Cost-effective and Key to Hepatitis C Virus Elimination

**DOI:** 10.1093/ofid/ofad637

**Published:** 2023-12-18

**Authors:** Sophy T F Shih, Jack Stone, Natasha K Martin, Behzad Hajarizadeh, Evan B Cunningham, Jisoo A Kwon, Colette McGrath, Luke Grant, Jason Grebely, Gregory J Dore, Andrew R Lloyd, Peter Vickerman, Georgina M Chambers

**Affiliations:** The Kirby Institute, University of New South Wales, Sydney, New South Wales, Australia; Population Health Sciences, University of Bristol, Bristol, United Kingdom; Division of Infectious Diseases and Global Public Health, University of California San Diego, San Diego, California, USA; The Kirby Institute, University of New South Wales, Sydney, New South Wales, Australia; The Kirby Institute, University of New South Wales, Sydney, New South Wales, Australia; The Kirby Institute, University of New South Wales, Sydney, New South Wales, Australia; Justice Health and Forensic Mental Health Network, New South Wales Health, Sydney, New South Wales, Australia; Corrective Services New South Wales, Sydney, New South Wales, Australia; The Kirby Institute, University of New South Wales, Sydney, New South Wales, Australia; The Kirby Institute, University of New South Wales, Sydney, New South Wales, Australia; The Kirby Institute, University of New South Wales, Sydney, New South Wales, Australia; Population Health Sciences, University of Bristol, Bristol, United Kingdom; National Perinatal Epidemiology and Statistics Unit, Centre for Big Data Research in Health, University of New South Wales, Sydney, New South Wales, Australia

**Keywords:** DAA, economic evaluation, HCV, justice health, modeling

## Abstract

**Background:**

The Surveillance and Treatment of Prisoners With Hepatitis C (SToP-C) study demonstrated that scaling up of direct-acting antiviral (DAA) treatment reduced hepatitis C virus (HCV) transmission. We evaluated the cost-effectiveness of scaling up HCV treatment in statewide prison services incorporating long-term outcomes across custodial and community settings.

**Methods:**

A dynamic model of incarceration and HCV transmission among people who inject drugs (PWID) in New South Wales, Australia, was extended to include former PWID and those with long-term HCV progression. Using Australian costing data, we estimated the cost-effectiveness of scaling up HCV treatment in prisons by 44% (as achieved by the SToP-C study) for 10 years (2021–2030) before reducing to baseline levels, compared to a status quo scenario. The mean incremental cost-effectiveness ratio (ICER) was estimated by comparing the differences in costs and quality-adjusted life-years (QALYs) between the scale-up and status quo scenarios over 40 years (2021–2060) discounted at 5% per annum. Univariate and probabilistic sensitivity analyses were performed.

**Results:**

Scaling up HCV treatment in the statewide prison service is projected to be cost-effective with a mean ICER of A$12 968/QALY gained. The base-case scenario gains 275 QALYs over 40 years at a net incremental cost of A$3.6 million. Excluding DAA pharmaceutical costs, the mean ICER is reduced to A$6 054/QALY. At the willingness-to-pay threshold of A$50 000/QALY, 100% of simulations are cost-effective at various discount rates, time horizons, and changes of treatment levels in prison and community.

**Conclusions:**

Scaling up HCV testing and treatment in prisons is highly cost-effective and should be considered a priority in the national elimination strategy.

**Clinical Trials Registration:**

NCT02064049.

## Teaser

As the prison sector is a key venue for HCV transmission among people who inject drugs, broad implementation of direct-acting antiviral therapy in prisons is both clinically effective and cost-effective to be considered a priority in the national elimination strategy.

Globally, an estimated 57 million people have chronic hepatitis C virus (HCV) infection; notably, testing and linkage to care and treatment in vulnerable populations remain low [1]. People who inject drugs (PWID) comprised an estimated 8.5% of all HCV infections, and it is predicted that 79% of HCV infection in high-income countries would be prevented if transmission due to injecting drug use were removed [2]. There is a disproportionately high HCV prevalence in PWID in correctional settings, estimated at 15% of the 10 million people who are incarcerated at any one time worldwide [3]. The advent of direct-acting antivirals (DAAs) has revolutionized HCV therapy with cure rates >95% [4]. The broad implementation of DAA therapy has considerable public health potential, with the World Health Organization setting ambitious HCV elimination targets, including 90% reduction in HCV incidence, 80% of the infected population treated, and 65% reduction in HCV mortality by 2030 [5]. The key role the prison sector plays as a venue for HCV transmission primarily among PWID has led to prioritization of DAA treatment scale-up in Australian prisons [6].

The Surveillance and Treatment of Prisoners With Hepatitis C (SToP-C) study, a nonrandomized clinical trial within a longitudinal cohort of 3691 prisoners, evaluated the impact of DAA treatment on HCV incidence in 4 prisons located in Australia's most populous state, New South Wales (NSW) [7]. In the initial phase (pre-DAA treatment) from October 2014 to mid-2017, HCV treatment was delivered as the standard of care by the nurse-led prison-based hepatitis service, initially with interferon-based treatments and then with DAAs after the universal access to all Australians, including prisoners, from early 2016 [8]. In the second phase, commencing in mid-2017, intensive scale-up of testing and DAA treatment by increasing the capacity of prison health services with dedicated nurses to deliver HCV treatment and prison officers to facilitate prisoner movement was initiated and continued until study closure in November 2019. All 4 prisons offered some harm reduction services, including opioid agonist therapy (OAT). The SToP-C study demonstrated that rapid HCV treatment scale-up in prison reduced HCV transmission, with HCV incidence (primary and reinfection) being halved (adjusted hazard ratio, 0.50 [95% confidence interval {CI}, .33–.76]) [7].

Our previous mathematical modeling studies have shown the potential and importance of prison-based interventions for achieving HCV elimination for at-risk population in prisons and in the community [9, 10]. In the present study, we aimed to evaluate the cost-effectiveness of scaling up prison-based HCV testing and DAA treatment programs for PWID across NSW prisons with the impacts considered across both custodial and community settings, drew from empirical data of the costs and outcomes in the SToP-C study.

## METHODS

The economic evaluation was undertaken from a payer perspective considering costs incurred by the healthcare system and prisons, and health-related outcomes for current and former PWID in prisons and in the community. The population included in this modeling study was all people with ongoing or historic injecting drug use in the prison and community settings in NSW, Australia.

### Intervention and Comparator

The SToP-C testing and treatment intervention included enhanced, 6-monthly HCV surveillance among all adults aged ≥18 years incarcerated in 4 NSW prisons with increased capacity of the prison health services. All inmates with detectable HCV RNA underwent nurse-led pretreatment clinical and laboratory assessments and transient fibro-elastography (FibroScan). Infected participants eligible for HCV treatment were offered sofosbuvir/velpatasvir (400 mg/100 mg) once daily for 12 weeks, and those reinfected were offered retreatment. Participants transferred to non-SToP-C prisons continued DAA treatment, and those released to freedom were provided with continuing therapy and referral to community practitioner for follow-up. A detailed description of the SToP-C study has been published previously [7].

The intervention evaluated in this study was a scenario in which HCV treatment rate was scaled up in all NSW prisons by 44% (as achieved in the SToP-C prisons compared to other NSW prisons at that time [2017–2019]) for 10 years (from 2020 to 2030) but was then reduced to baseline (standard of care/business as usual at 2020 levels). The 44% increase was estimated by comparing the number of treatments per 100 inmates in the SToP-C prisons to the number of treatments in the non-SToP-C prisons for the period mid-2017 to the end of 2019. The scale-up scenario of HCV treatment in prisons in this study referred to increasing the coverage of the SToP-C HCV testing and DAA treatment to correctional facilities statewide in NSW.

The comparator was a counterfactual status quo scenario in which business as usual or standard of care in prison and community assumed that existing pathways of HCV testing and DAA treatment in prisons continued at 2020 levels (1500 treatments in 2020 with 32.9%–83.3% among PWID). This entailed targeted HCV screening on entry to a prison for those who reported injecting drug use, followed by a nurse-led model of care for further clinical and laboratory assessment with a small subset being triaged for specialist care [8]. After completion of the SToP-C study in 2019, considerable increase in DAA treatment has since occurred in NSW prisons and the treatment level in 2020 was a reflection of what if no further investment in prison HCV testing and treatment efforts occurred. This comparator choice of counterfactual to the scale-up of the HCV testing and DAA treatment as in the SToP-C study model by boosting capacity of the prison health services, with funding of dedicated nurses to deliver HCV treatment and dedicated prison officers to facilitate prisoner movement, was to evaluate the additional health benefits generated by the increased level of investment in prison HCV services.

### Mathematical Model

**Figure 1.**
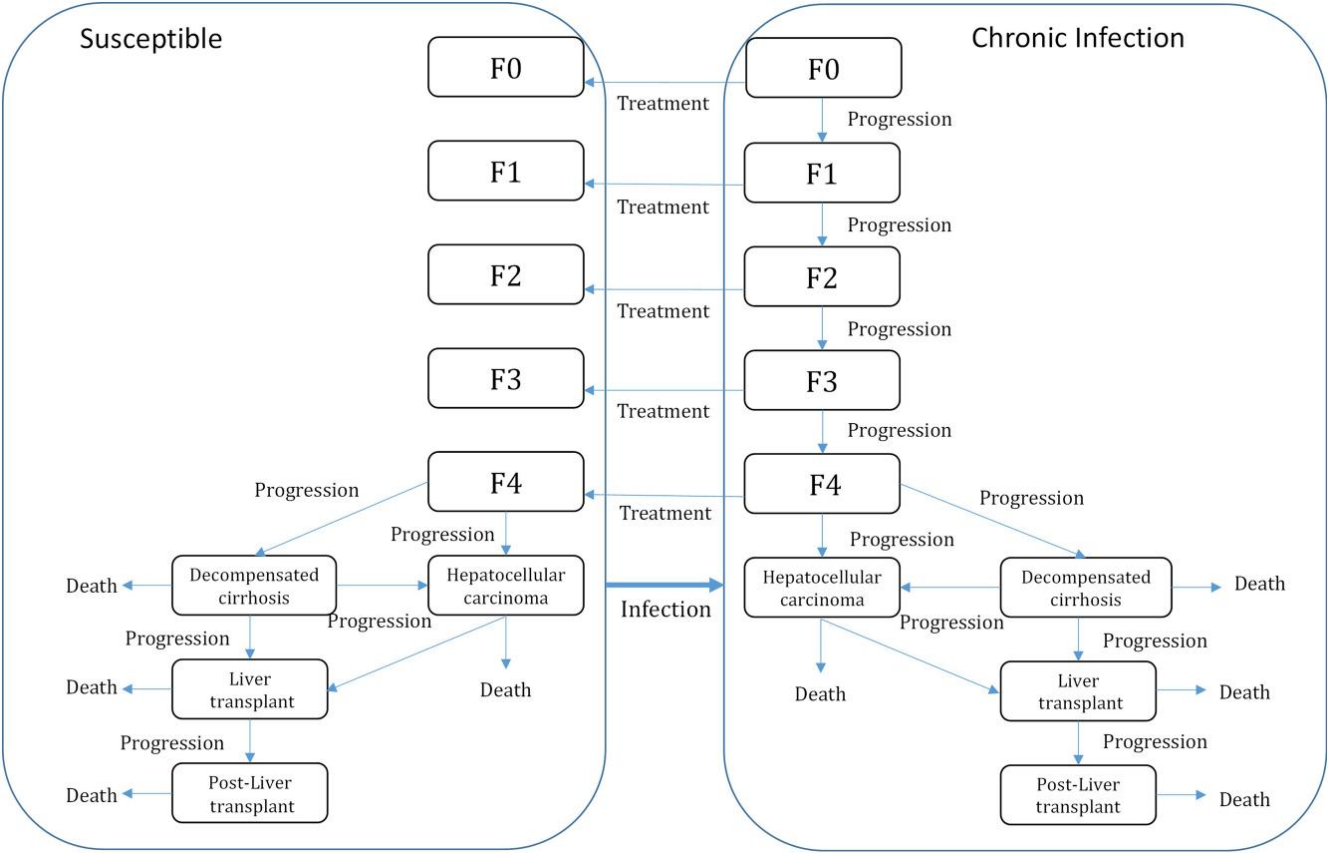
Model schematics of chronic hepatitis C progression with disease states.

Source: Sweeney et al [11].

We extended our published dynamic, deterministic model of incarceration and HCV transmission among current PWID in NSW prisons to include the HCV disease progression (shown in Figure 1, parameters shown in the Supplementary Materials Table S8) and mortality among PWID who ceased injecting [12]. In brief, the model stratified current PWID by injecting duration, incarceration status, HCV infection and disease status, and OAT status. Additionally, those who ceased injecting drugs were stratified by HCV infection and disease status. The model included HCV transmission among current PWID only, both in prison and the community. Transmission occurred at a rate proportional to chronic HCV prevalence in each setting. These rates differed by setting (prison or community), with elevated rates among PWID with <3 years of injecting [13, 14] or who had been recently released from prison [15], while rates were reduced if PWID were on OAT [16]. Most new infections without treatment led to chronic infection [17]. Current and former PWID with HCV infection could be treated at differing rates, which also varied for current PWID by setting, with community PWID on OAT being more likely to be treated than those not on OAT [18, 19]. A high proportion of treated individuals achieved sustained virological response. Those failing treatment or reinfected following successful treatment were eligible for retreatment due to unrestricted access to DAAs in Australia. Key model parameters are summarized in Table 1. The model was previously parameterized, calibrated, and validated using epidemiological and HCV treatment data in NSW using an approximate Bayesian computation sequential Monte Carlo algorithm [9]. We utilized previous 1000 model fits to project the long-term HCV morbidity and mortality.

**Table 1.** Summary of Key Epidemiological Data/Parameters

Data/Parameter	Value	Source/Notes
Number of PWID in the community	26 500–46 000	Larney et al, 2017 [20]
Mortality rate out of OAT (per 1000 person-years)	8.9 (95% CI, 8.6–9.2)	NSW data; Degenhardt et al, 2009 [21]
Relative risk of all-cause mortality if on OAT	0.33 (95% CI, .28–.39)	Degenhardt et al, 2019 [22]
% of community PWID who have ever been incarcerated	52% (range: 49.9%–53.9%)	Heard et al, 2020 [23]. Model is calibrated to the proportion of community PWID who have ever been incarcerated by duration of injecting; proportion of incarcerated PWID who are incarcerated for first time by duration of injecting (see [12]).
% of community PWID who have recently been incarcerated (<12 mo)	14% (range: 12.9%–15.7%)	
Average duration of each incarceration (mo)	Uniform with range 4–6	Mean length of adult incarceration episodes among opioid-dependent people who have ever been registered for OST is 5.1 mo. Assume average duration for PWID is similar with additional uncertainty.
Anti-HCV prevalence in community PWID	45% (range: 43.5%–47.4%)	Heard et al, 2020 [23]. Model is calibrated to HCV antibody prevalence (2010–2018) among community PWID by incarceration history.
Relative increase in HCV transmission risk among recently released PWID (12 mo since release)	2.78 (95% CI, 2.00–3.85)	Australian studies from systematic review [15]
Proportion of new infections that spontaneously clear	0.26 (95% CI, .22–.29)	Micallef et al, 2006 [17]
% of PWID in the community who have ever been treated	64% (95% CI, 60.4%–67.8%)	Heard et al, 2020 [23]. Model calibrated to trend over 2010–2018.
Number of HCV treatments in prison per year (2018–2020)	1500	Assume all treatments ever are among people with a history of IDU and these are given randomly between those with recent or ever IDU (see proportion in next row)
Proportion of HCV treatments in prison among current PWID	Range: 32.9%–83.3%	Range based on the proportion of prisoners with a history of IDU who recently injected drugs before their incarceration. [Drug use in prison surveys and prisoner health surveys]
Average annual treatment rate in former PWID	38%	Proportion of former PWID (distant drug dependence without drug use >12 mo) received DAA (2016–2018)
Effect of intervention on the rate of HCV treatment in prison	44%	Treatment rate in SToP-C prisons to be 44% higher than in non-SToP-C prisons [7]

Full description of the model is available in [12].

Abbreviations: CI, confidence interval; DAA, direct-acting antiviral; HCV, hepatitis C virus; IDU, injecting drug use; NSW, New South Wales; OAT, opioid agonist therapy; PWID, people who inject drugs; SToP-C, Surveillance and Treatment of Prisoners With Hepatitis C study.

### Quality of Life Utility

Quality-of-life (QoL) utility weights were stratified by prison and community, injecting drug use status (current and former PWID), and infection status (never infected and ever infected at different HCV disease states). We used real-world QoL data collected using the EQ-5D-5L (EuroQol Research Foundation) survey tool from 2 Australian studies: the enhancing treatment of hepatitis C in opioid substitution (ETHOS) Engage study for PWID in the community and the SToP-C study for prisoners [7, 24]. The ETHOS Engage study was an observational cohort study of 2395 PWID attending drug treatment clinics and needle syringe programs in Australia. HCV infection and disease stages among the participants of the ETHOS Engage and SToP-C studies included never infected and those infected at chronic hepatitis stage of F0 to F4, with very few having cirrhosis or progressed liver diseases. Therefore, for the later stages of chronic hepatitis, a systematic review of QoL estimates for HCV infections was used to approximate the utility values through estimating detriment factor (dis-utility), based on differences in the utility weights for disease states F0/F1 between our data and the systematic review [25] (Table 2). Health utilities were summed for the modeled population over the time horizon (discounted at 5% per annum in base-case analyses as recommended for Australia) to generate quality-adjusted life-years (QALYs) as the primary health outcome [27].

**Table 2.** Health-Related Quality of Life Utilities of People Who Inject Drugs in the Community and People at Risk of Hepatitis C Virus Infection in Prison

Disease Status	PWID in Community		People at Risk of HCV in Prison	
	QoL Utility (95% CI)	Source/Notes	QoL Utility (95% CI)	Source/Notes
Never infected	0.771 (.751–.791)	ETHOS Engage wave 1 & 2 EQ-5D-5L	0.931 (.924–.939)	SToP-C. Data suggest same utilities for never infected and F0–F4.
F0/F1	0.74 (.733–.764)	ETHOS Engage wave 1 & 2 EQ-5D-5L	0.931 (.924–.939)	SToP-C. Data suggest same utilities for never infected and F0–F4.
F2	0.753 (.719–.787)	ETHOS Engage wave 1 & 2 EQ-5D-5L	0.931 (.924–.939)	SToP-C. Data suggest same utilities for never infected and F0–F4.
F3	0.734 (.674–.793)	ETHOS Engage wave 1 & 2 EQ-5D-5L	0.931 (.924–.939)	SToP-C. Data suggest same utilities for never infected and F0–F4.
F4/compensated cirrhosis	0.716 (.662–.771)	ETHOS Engage wave 1 & 2 EQ-5D-5L	0.931 (.924–.939)	SToP-C. Data suggest same utilities for never infected and F0–F4.
Decompensated cirrhosis	0.591 (.536–.645)	0.657 (.602–.711) from [25] with detriment based on difference in utility for F0/F1: 0.066 between ETHOS and [25]	0.782 (.727–.836)	0.657 (.602–.711) from [25] with increment based on difference in utility for F0/F1: 0.125 between SToP-C and [25]
Hepatocellular carcinoma	0.651 (.581–.722)	0.717 (.647–.788) from [25] with detriment based on difference in utility for F0/F1: 0.066 between ETHOS and [25]	0.842 (.772–.913)	0.717 (.647–.788) from [25] with increment based on difference in utility for F0/F1: 0.125 between SToP-C and [25]
Liver transplant	0.434 (.334–.624)	0.50 (.40–.69) from [26] with detriment based on difference in utility for F0/F1: 0.066 between ETHOS and [25]	0.625 (.425–.815)	0.50 (.40–.69) from [26] with increment based on difference in utility for F0/F1: 0.125 between SToP-C and [25]
Post–liver transplant	0.646 (.591–.701)	0.712 (.657–.767) from [25] with detriment based on difference in utility for F0/F1: 0.066 between ETHOS and [25]	0.837 (.782–.892)	0.712 (.657–.767) from [25] with increment based on difference in utility for F0/F1: 0.125 between SToP-C and [25]

Abbreviations: CI, confidence interval; EQ-5D-5L, EuroQol Research Foundation survey tool; ETHOS, enhancing treatment of hepatitis C in opiod substitution; HCV, hepatitis C virus; IDU, injecting drug use; PWID, people who inject drugs; QoL, quality of life; SToP-C, Surveillance and Treatment of Prisoners With Hepatitis C study.

### Cost Assessment

Costs were estimated for HCV screening, diagnosis, treatment, and chronic HCV–related care (Table 3). Costs were discounted at 5% per annum and reported in 2021 Australian dollars (A$) from a payer perspective (ie, the Australian government and the State Justice Health). HCV diagnosis costs were estimated as the weighted average of different testing strategies in the community and prisons. Pathway analyses were conducted for each testing strategy considering clinical staff time (eg, general practitioner, nurse, phlebotomist), correctional officer time (prison only), and test costs [28]. The average pharmaceutical cost of DAA treatment was estimated based on the analysis of Pharmaceutical Benefits Scheme (PBS) claimed data for HCV DAA treatment [29], despite at the time of the SToP-C study that a fixed contract for supply of DAA has been agreed between the Australian government and the manufacturer. Dispensing costs were calculated by the PBS dispensing fee schedule in the community and estimated by an ingredient-based analysis (considering capitals, labor, consumables, overheads, and custodial management) for DAA treatment in prisons. Monitoring regimens during and after treatment completion followed the standard of care (3 visits) in prison and community.

**Table 3.** Model Inputs of Cost Estimates (2021 Australian Dollars, A$)

Costs	Value With Uncertainty Range, A$	Notes
Average cost of antibody test		
PWID in prison	$57 ($52–$66)	Cost weighted across pathways^a^
PWID in community	$68 ($56–$90)	Cost weighted across pathways^a^
Former PWID in community	$83 ($62–$120)	Cost weighted across pathways^a^
Average cost of HCV RNA test		
PWID in prison	$110 ($101–$125)	Cost weighted across pathways^a^
PWID in community	$119 ($105–$145)	Cost weighted across pathways^a^
Former PWID in community	$132 ($110− $170)	Cost weighted across pathways^a^
Unit costs of HCV treatment and diagnosis		
DAA pharmaceutical costs	$10 887 ($7098–$16 260)	Uncertainty based on ± 10%
DAA dispensing costs in prison	$483 ($435–$531)	Uncertainty based on ± 10%
HCV care delivery and monitoring costs in prison		
Standard of care (business as usual)	$570 ($541–$605)	Uncertainty based on ± 10%
Scale-up of DAA treatment	$1107 ($1058–$1195)	Uncertainty based on ± 10%
HCV care delivery and monitoring costs in community		
PWID	$650 ($585–$715)	Uncertainty based on ± 10%
Former PWID	$681 ($613–$749)	Uncertainty based on ± 10%
DAA dispensing costs in community	$504 ($454–$555)	Uncertainty based on ± 10%
Annual costs of HCV disease–related care		
F0–F3	$172 ($143–$434)	Medical management of chronic infection^a^
F4/Compensated cirrhosis	$1450 ($1438–$2060)	Medical management of chronic infection^a^
Decompensated cirrhosis	$10 093 ($9084–$11 102)	Medical management of chronic infection^a^
Hepatocellular carcinoma	$74 361 ($66 925− $81 797)	Medical management of chronic infection^a^
Liver transplant	$107 433 ($96 690–$118 176)	Medical management of chronic infection^a^
Post–liver transplant	$4721 ($4249–$5193)	Medical management of chronic infection^a^

Abbreviations: A$, Australian dollars; DAA, direct-acting antiviral; HCV, hepatitis C virus; PWID, people who inject drugs.

^a^Details are shown in the Supplementary Materials.

Annual costs of HCV disease-related care were assessed for disease states F0–F3, compensated cirrhosis (F4), decompensated cirrhosis, hepatocellular carcinoma, liver transplant, and posttransplantation. For each disease state, annual costs were estimated for related care including consultations, pathology tests, medications, examination procedures (eg, ultrasound, endoscopy, paracentesis, transarterial chemoembolization), and surgery. Unit costs were sourced from the PBS [30], the Medicare Benefits Schedule [31], the Diagnostic Related Group weights published by the Australian Independent Hospital Pricing Authority [32], and public salary numeration scales for nurses and correctional officers [33, 34]. Details are presented in the Supplementary Materials Tables S3–S7.

### Cost-effectiveness Analysis

We estimated the cost-effectiveness of scaling up prison-based testing and treatment by comparing the scale-up scenario of increasing HCV treatment by 44% for 10 years (from 2021 to 2030) and then reducing HCV testing and treatment to baseline levels with a counterfactual (business as usual) scenario assuming the treatment uptake as in 2020. For both scenarios, existing levels of treatment in the community were assumed to continue at current rates (for PWID and former PWID). By comparing the difference in costs of testing/treatment and HCV care in the community/prison and changes in QALYs between the scale-up and business as usual scenarios over 2021 to 2060 (time horizon 40 years), we estimated the mean incremental cost-effectiveness ratio (ICER). Unit costs and QoL utilities were sampled from probability distributions (Tables 2 and 3 and applied to each of the 1000 model fits [10]. The mean ICER was estimated in terms of the mean (across 1000 model runs) discounted incremental costs divided by the mean discounted incremental QALYs averted. Probabilistic sensitivity analysis (PSA) using Monte Carlo simulation was performed to generate cost-effectiveness planes and to derive 95% credible intervals (CrIs). The ICER was compared to a A$50 000 per QALY willingness-to-pay (WTP) threshold [35]. Cost-effectiveness acceptability curves were plotted to determine the proportion of model simulations that are cost-effective as a function of WTP thresholds.

### Sensitivity Analysis

The variations in 1000 model fits based on sampled QoL utilities and costs were used to conduct a PSA. We also performed the following multiple univariate sensitivity analyses to test the impact of assumptions on the ICER including (1) time horizon of 20 years (baseline 40 years); (2) discount rates of 0%, 3%, or 7% (baseline 5%); (3) removing the cost of DAAs; (4) scenarios in which community treatment uptake (PWID and former PWID) were either increased or reduced by 25% from 2021 onward; (5) a lower (25%) or higher (75%) scale-up in the prison-based HCV treatment uptake over 2021–2030 (baseline: 44%); and (6) reinfection risk that is lower (by 50%) or higher (double) the risk of primary infection (baseline: same risk).

### Uncertainty Analysis

For our baseline analyses, we performed a regression analysis of covariance to determine which parameter uncertainties contribute most to variability in the ICER, incremental costs, and QALYs.

## RESULTS

Maintaining HCV treatment uptake levels at 2020 would cost A$369.5 million (discounted) over 40 years (2021–2060). In contrast, scaling up HCV treatment across NSW prisons by 44% for 10 years (2021–2030) and then reducing to business as usual would cost A$373.1 million (discounted 5%) for the same period. Scaling up HCV treatment in prison for 10 years would incur an additional A$4.1 million in testing and treatment costs but would save A$0.6 million in HCV-related care costs. Overall, the scale-up of prison-based HCV treatment would result in a mean net incremental cost of A$3.6 million (Table 4).

**Table 4.** Cost-effectiveness of Scaling up Hepatitis C Virus Treatment of the Base-Case Analyses With a Time Horizon of 40 Years and a Discount Rate of 5% per Annum

Costs	Scaled-up Prison-Based HCV Treatment Over 2021–2030	Business as Usual	Incremental
Cost of HCV screening and treatment (in million A$; 2021–2060)	135.6	131.5	4.1
Cost of HCV disease–related care (in million A$; 2021–2060)	237.5	238.1	−0.6
Total costs (in million A$; 2021–2060)	373.1	369.5	3.6
QALYs	1 106 292	1 106 018	275
Mean ICER (A$ per QALY gained)	…	…	12 968

Abbreviations: A$, Australian dollars; HCV, hepatitis C virus; ICER, incremental cost-effectiveness ratio; QALY, quality-adjusted life-year.

**Figure 2.**
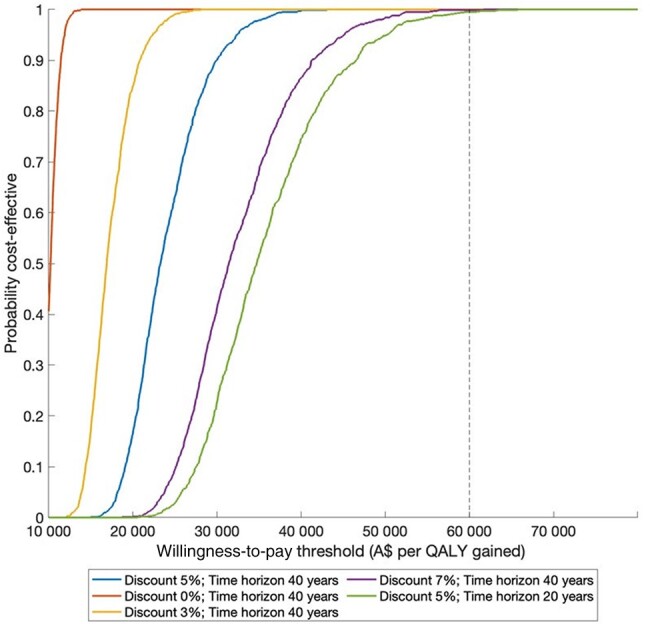
Cost-effectiveness acceptability curve for the base-case cost-effectiveness analysis and different discount rates and time horizons. The dashed vertical line shows the willingness-to-pay threshold of A$50 000 per quality-adjusted life-year (QALY) gained.

**Figure 3.**
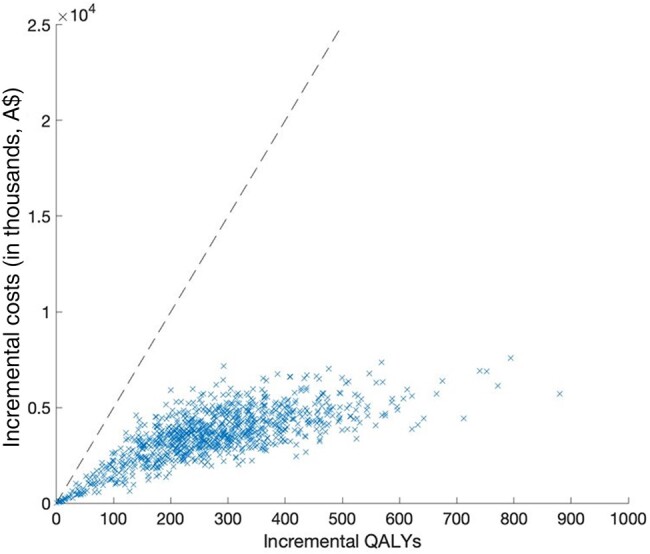
Cost-effectiveness plane for the baseline cost-effectiveness analysis. Blue crosses represent each model run. The dashed line shows the willingness to-pay threshold of A$50 000. Abbreviation: QALY, quality-adjusted life-year.

Over the 40-year time horizon in the base case, scaling up HCV treatment in prison by 44% for 10 years would gain 274.5 QALYs (discounted) by preventing infected cases from progressing to late-stage liver disease and preventing 6.6% (95% CrI, .7%–8.5%) new HCV infections (“treatment as prevention effect”) over the time period 2021–2060. Therefore, scaling up the prison-based HCV treatment is projected to have a mean ICER A$12 968/QALY gained. At the WTP threshold of A$50 000/QALY, the cost-effectiveness acceptability curve indicates that 100% simulated iterations are cost-effective under various discount rates and time horizons (Figure 2). Uncertainty of the baseline analysis is illustrated in the cost-effectiveness plane by the PSA showing that all simulated iterations are below A$50 000/QALY (Figure 3).

**Figure 4.**
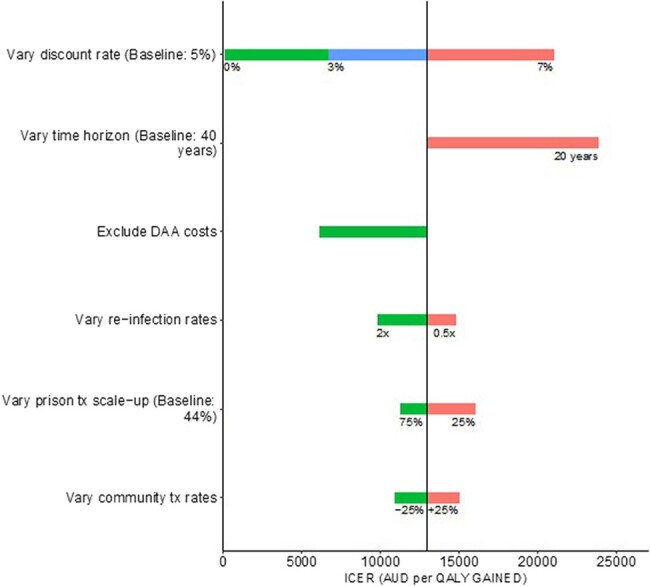
Sensitivity analyses on various assumptions. Red, blue, and green bars show the mean incremental cost-effectiveness ratio (ICER) in each of the sensitivity analyses. The solid black line shows the mean baseline ICER of A$12 968 per quality-adjusted life-year. Abbreviations: DAA, direct-acting antiviral; ICER, incremental cost-effectiveness ratio; QALY, quality-adjusted life-year; Tx, treatment.

The most significant factor impacting ICER results is the discount rate; at an annual 7% discount rate the ICER is A$21 017/QALY compared to A$52.4/QALY if costs and QALYs are undiscounted. Scaling up HCV treatment in prison would still be highly cost-effective (ICER A$23 879/QALY) over a shorter time horizon of 20 years (vs 40 years in the base case). When excluding the DAA medication cost, the ICER is markedly reduced to A$6054/QALY (discounted 5%). Varying the treatment scale-up in prison and treatment level in community does not change the results significantly (ICER A$10 891–A$16 035/QALY; Figure 4) with the prison scale-up of treatment being more cost-effective if a greater scale-up in prison is achieved or if community treatment rates are reduced. Varying the risk of HCV reinfection following treatment does not affect the results significantly (ICER A$9755–A$14 770), with scale-up of HCV treatment in prison being more cost-effective if reinfection rates are higher.

In baseline analyses, most of the variability in the ICER and incremental QALYs were due to uncertainty in parameters defining rates of progression from F0 to compensated cirrhosis (combined: 47% of variability) and the HCV chronic prevalence in prisons (31% of variability). Uncertainty in the HCV chronic prevalence in prisons (49% of variability) and the community (5% of variability), DAA drug costs (18% of variability), and the PWID population size (7% of variability) contributed most of the variability in the incremental costs.

## DISCUSSION

More than half of the SToP-C participants (n = 1926/3691) reported a history of injecting drug use, and 22% (n = 797/3691) reported injecting in the past month with the majority (91%, 727/797) reporting sharing injecting equipment in prison [7]. The risk of acquiring HCV in the correctional setting is disproportionately higher than other venues, so that mitigation of transmission risk by timely detection and treatment is critical to reduce the burden of HCV and achieve HCV elimination goals. Our modeling results based on key SToP-C study parameters suggest that scaling up of HCV treatment in correctional facilities is highly cost-effective with all ICERs below the common WTP threshold A$50 000/QALY.

Five studies, from the United Kingdom, United States, Spain, Ireland, and Australia, all based on modeling simulations, reported cost-effectiveness of scaling up DAA treatment for HCV in prisons [36–40]. All studies showed that DAA treatment scale-up in prisons is cost-effective and pivotal to HCV elimination target. Targeting on PWID or combined with addiction services (eg, OAT, needle syringe program) would further improve the cost-effectiveness of prison DAA treatment. Our current modeling adds to these studies by demonstrating that a scale-up of prison-based treatment is still cost-effective in a setting with high levels of existing treatment (in prison and community); and further scale-up of prison HCV treatment will be important if community treatment rates fall.

Strengths of the present study include use of real-world evidence from the SToP-C intervention effectiveness and patient-level QoL data collected from the SToP-C participants in prisons and PWID in the community. However, our study also reveals the challenges in patient-reported outcome from this marginalized population that was predominantly young male prisoners (82% male; median age, 33 years). The QoL measured by the EQ-5D-5L in the SToP-C incarcerated participants showed relatively high QoL utility scores for those with negative HCV antibody (“never infected”) (0.931 [95% CI, .924–.939]). Across all chronic hepatitis stages and infection status, the QoL was higher in prisoners than their counterparts in the community, using the same EQ-5D-5L instrument in the community survey, ranging from 0.771 (95% CI, .751–.791) in those never infected to 0.716 (95% CI, .662–.771) in those with compensated cirrhosis (F4). It is unclear what contributes to this apparent QoL discrepancy between the prison and community, but it could be due to prisoners disregarding (discounted) their circumstances when completing the EQ-5D-5L, or that participants in the community cohort were older (55% male; median age, 44 years) compared to prisoners [19]. Contrary to the findings from studies in the correctional setting [41], the reported QoL among the SToP-C participants is comparable to the population norm (mean EQ-5D-5L, 0.95 for males aged 25–34 years) [42]. Our previous study of QoL among PWID suggests that 2 factors (ie, having stable housing and being employed), are positively correlated with better QoL [43, 44]. The population of PWID, dominantly young males (median age, 43 years; 67% male), generally reported little problems in mobility, self-care, and usual activities but experienced pain/discomfort and anxiety/depression. Interestingly, the status of HCV infection, liver disease staging, currently on OAT, or high-risk alcohol consumption showed little impact on health-related QoL. Even in people who were treated with DAA, few changes in QoL were observed following completion of treatment throughout follow-up at end of study in the participants across North America, Europe, and Australasia [43].

A potential limitation of our analysis is that the modeled intervention (scale-up of HCV treatment by 44%) was achieved with significant resources (such as a dedicated officer, nurse, medical doctor, and room space) that may not be attainable in routine care. Although the standard of care in NSW prisons has been changed, informed by the SToP-C study, findings from our current modeling study may be applicable to other settings internationally where prison-based HCV testing and treatment uptake is still low.

Determining the costs of DAA medication in Australia is complex. The listed reimbursement prices for sofosbuvir/velpatasvir (400 mg/100 mg tablet) are in the range of A$14 500–A$14 651 [30]. However, the fixed prices for DAA medicines published by the Australian PBS are not the true costs borne by the Australian government given a negotiated risk-sharing agreement with a complex rebate system. The Australian government provided at least A$1 billion investment in DAA treatment for HCV infection over 5 years from March 2016 regardless of the prescription quantity, but full details of the contracts are not available. What is clear is that the unit cost for 1 course of DAA treatment became progressively cheaper above a threshold number of prescriptions. We used A$10 887 in the base case and the range of A$7098–A$16 260 in the sensitivity analysis for 1 DAA treatment course based on the claimed DAA medicine statistics [29]. As the DAA pharmaceutical costs accounted a large proportion of overall costs (which also include follow-up monitoring, testing, and management), we tested the impact of the substantial cost by excluding the DAA medications in the sensitivity analysis. In the context of a fixed contract reimbursement system, additional DAA medications associated with scale-up of HCV treatment in the scenarios of our study are essentially sunk costs. Exclusion of DAA pharmaceutical cost is more appropriate in the Australian decision context. However, inclusion of DAA pharmaceutic costs would make our study results more generalizable to other settings where DAA pharmaceutical contract is different to the “subscription model” in Australia [45].

In conclusion, scaling up of HCV testing and treatment in prisons is not only effective in reducing HCV incidence but also cost-effective, compared to unchanged HCV services as at the 2020 level. The scale-up of HCV treatment efforts and resource should be considered a priority in the national elimination strategy.

## Supplementary Material

ofad637_Supplementary_Data

## Supplementary Data

Supplementary materials are available at *Open Forum Infectious Diseases* online. Consisting of data provided by the authors to benefit the reader, the posted materials are not copyedited and are the sole responsibility of the authors, so questions or comments should be addressed to the corresponding author.

## Notes

***Author contributions.*** A. R. L., G. J. D., J. S., N. K. M., P. V., and G. M. C. conceived the study and developed analysis plan. J. S. adapted the model and performed model analyses. S. T. F. S., J. A. K., and G. M. C. developed cost estimates. S. T. F. S., E. B. C., and J. G. conducted analysis of quality of life data and developed utility estimates. B. H., E. B. C., C. M., L. G., J. G., G. J. D., and A. R. L. provided study materials and data. S. T. F. S., J. S., B. H., E. B. C., J. G., A. R. L., and G. M. C. analyzed and interpretated data. S. T. F. S. drafted the manuscript and all authors critically reviewed and approved the manuscript.

***Patient consent.*** This modeling study does not include factors necessitating patient consent as no patient-level data were used in the analysis.

***Data availability.*** Model input parameters are presented in Table 1 and the Supplementary Materials Table S8. Further details will be made available to others upon request. Model code will be shared with researchers who provide a methodologically sound proposal approved by J. S. and P. V.

***Financial support*.** This work was supported by the National Health and Medical Research Council (Award number APP1092547) in partnership with Gilead Sciences. A. R. L. was supported by an National Health Medical Research Council Practitioner Fellowship (award number APP1137587). G. J. D. is funded by an National Health Medical Research Council Investigator Grant (grant number 2008276). J. S. and P. V. acknowledge funding from the Wellcome Trust (grant number 226619/Z/22/Z) and the National Institute for Health and Care Research Health Protection Research Unit in Behavioural Science and Evaluation at the University of Bristol. For the purpose of Open Access, the author has applied a CC BY public copyright licence to any Author Accepted Manuscript version arising from this submission.
