# Supplementary material for: Scale-up of Direct-Acting Antiviral Treatment in Prisons Is Both Cost-effective and Key to Hepatitis C Virus Elimination
Source: Open Forum Infect Dis. 2023 Dec 18;11(2):ofad637. doi: 10.1093/ofid/ofad637 (PMC10854215; doi:10.1093/ofid/ofad637)
Supplement: ofad637_Supplementary_Data [file ofad637_supplementary_data.pdf]

Supplementary Materials

Title: Scale-up of direct-acting antiviral treatment in prisons is both cost-effective and key to HCV elimination

Sophy TF Shih[1*] (DrPH), Jack Stone[2*] (PhD), Natasha Martin[3] (PhD), Behzad Hajarizadeh[1] (PhD), Evan Cunningham[1] (PhD), Jisoo A Kwong (PhD), Colette McGrath[4] (MHM), Luke Grant[5] (BA), Jason Grebely[1] (PhD), Gregory J Dore[1] (PhD), Andrew R Lloyd[1] (PhD), Peter Vickerman[2] (DPhil), Georgina M Chambers[6] (PhD)
*Joint first authors
1. The Kirby Institute, UNSW Sydney, NSW 2052, Australia;
2. Population Health Sciences, University of Bristol, Bristol, UK;
3. Division of Infectious Diseases & Global Public Health, University of California San Diego, San Diego, USA;
4. Justice Health and Forensic Mental Health Network, NSW Health, Sydney, Australia;
5. Corrective Services NSW, Sydney, Australia;
6. National Perinatal Epidemiology and Statistics Unit, Centre for Big Data Research in Health, UNSW Sydney, NSW 2052, Australia

Table of Contents

1. Costing for HCV testing 2
2. Costing of DAA treatment and monitoring 4
3. Costing of chronic hepatitis C management 6
4. Disease Progression Parameters 9

1. Costing for HCV testing

The testing pathways for HCV require an antibody test for exposure followed by an RNA test for confirmation of active infection. The testing pathways involve different professional staff by different approach in different settings. Table S1 presents the testing pathways and their activities and various staff (coloured) who could attend for these activities.
HCV diagnosis costs were estimated by the weighted average of different testing strategies in the community and prison, based on the testing and treatment occurred in community health centres, drug centres general practice, specialist clinics, and other settings from the REACH study, a national observational cohort that includes 33 diverse study sites from ACT, NSW, NT, QLD, SA, TAS, VIC and WA.[1] The numbers of patients by injecting drug use status were used to estimate the weighted average of current PWID and former PWID. The weighted average costs in former PWID higher than those of current PWID were due to higher proportion of patients cared by specialists who charge higher costs.

Table S1: Schedule of assessments in HCV testing prior to DAA treatment

Test & staff involved	Initial presentation	Review of HCV antibody test result	Review of HCV RNA test result	Assessment of test results	Prescription of DAA treatment
Nurse (RN) 5 years exp	X/X	X/X			
Nurse (CNS)			X/X	X/X	
Nurse (CNC)					X
Physician (General Practitioner)	X/X	X	X		X
ID specialist		X	X		X/X
ID specialist (prison)					X (2%)*
Prison guard	X	X	X	X	X
Risk assessment (RN)	X				
Medical history (CNS)				X	
Vital signs & physical measurements (CNS)				X	
Phlebotomy (on-site)	X/X	X		X	
Pathology collection (off-site)	X				
HCV antibody testing	X				
HCV RNA testing (qualitative)		X			
Liver function tests				X	
Full blood count				X	
Biochemistry (Urea, Electrolytes, Creatinine) (U&E)				X	
Clotting (INR, Prothrombin time)				X	
HIV & HBV serology	X				
Pregnancy Test (serum or urine)				X (30%)*	
FibroScan	-	-	-	X (40%)*	-

X- Prison CNS+CNC, X- Prison CNS+ID, X- GP, X- Specialist, X – Testing * Percentage of participants applicable to the test and service

2. Costing of DAA treatment and monitoring

The scale-up of DAA treatment in prisons involves multiple (three) visits by dedicated nurses with presence of prison guards. The tests at each monitoring visit in different time points are presented in Table S2. Based on the SToP-C study protocol, additional three visits were scheduled.[2] Labour unit costs were based on Public Health System Nurses' and Midwives' (State) Award for nursing staff and Award of Industrial Relations Commission for correctional officers.[3] Pathology costs were sourced from the Australian Medical Benefits Scheme (MBS).[4]

The pharmaceutical costs of DAA treatment was based on the analysis of Pharmaceutical Benefits Scheme (PBS) claimed data, although a fixed contract (fixed costs irrespective of quantity consumed) for supply of DAA has been agreed between the Australian Government and the manufacture.[5] As the fixed contract involved negotiation of risk-sharing agreement with a complex rebate system between the pharmaceutical manufacturers and the Australian government, the true cost was difficult to estimate, but the PBS claimed data offered an indication of the DAA treatment costs for HCV in the context of Australian healthcare system.

DAA dispensing cost in prison was estimated by an ingredient-based costing, considering program management & administration, equipment, pharmacy staff, custodial staff, administration overhead (IT, finance, and payroll). DAA dispensing cost in the community was estimated by the cost items of PBS reimbursements to pharmacist including dispensing fee, maximum ex-manufacturer mark-up, Tier three Administration, Handling and Infrastructure (AHI) fee, and concession patient contribution.[6]

Table S2: Schedule of assessments post DAA treatment initiation

Test & staff involved	4 week*	8 week (Tx support visit)*	12week (end of Tx)*	24 wk (SVR12)	48-52 week (1 yr post Tx)
Nurse (CNS)	X	X	X	X	X
Prison guard	X	X	X	X	X
Phlebotomy (blood sample)	X		X	X	X
HCV RNA testing (quantitative)	X		X	X	X
Liver function tests	X		X	X	
Full blood count	X		X	X	
Biochemistry (Urea, Electrolytes, Creatinine) (U&E)	X		X	X	
HIV & HBV serology				X	
Pregnancy Test (serum or urine)	X		X		

* Additional visits in the SToP-C study

3. **Costing of chronic hepatitis C management**

Costs of chronic hepatitis C related care were updated from the previous study.36 Micro-costing methods were used to determine the quantities of healthcare services and annual costs of care for patients with HCV stages F0-F3 (medical management in subsequent years in Table S3), F4 (medical management for compensated cirrhosis in subsequent years in Table S4), decompensated cirrhosis/liver failure (healthcare resources required per annum in Table S5), hepatocellular carcinoma (healthcare resources required per annum in Table S6), liver transplant, and post-transplant care (healthcare resources required per annum in Table S7). Costs included specialist visits, clinical care nurse visits, pathology, medical procedures, and hospital admissions. Unit costs were obtained from the MBS (assuming 100% government benefits) for medical services, procedures, and pathology tests. Medication costs were sourced from the Australian Pharmaceutical Benefits Scheme (PBS).[4,6] Inpatient medical procedures and stays were sourced from the National Hospital Cost Data Collection using Australian Related fined Diagnosis Related Groups (AR-DRG).[7]

Table S3: Medical management of chronic infection (F0-F3) in sequent years following DAA treatment

	**DAA responder**	**DAA non-responder (switch to another DAA)**
**Health professionals' visits and tests**	**Qty per year**	**Qty per year**
Nurse (CNS)	1	2
Prison guard		2
Phlebotomy (blood sample)		2
HCV antibody testing	1	2
HCV RNA testing	1	2

Table S4: Medical management of compensated cirrhosis (F4) in sequent years following DAA treatment

	**DAA responder**	**DAA non-responder (switch to another DAA)**
**Health professionals' visits and tests**	**Qty per year**	**Qty per year**
Nurse (CNS)	1	6
Nurse (CNC)	1	
Prison guard		6
Phlebotomy (blood sample)	1	5
HCV RNA testing	1	4
HIV serology	1	1
Liver function test	2	5
Full blood count	2	5
Clotting (INR, Prothrombin time)	2	5
FibroScan		1
AFP	2	2
Abdominal ultrasound	2	2
Procedures		
Diagnostic endoscopy (+ anaesthetic)	0.5*	0.5*

* 1 in 2 patients need the endoscopic procedure

Table S5: Healthcare resources requirement for decompensated cirrhosis and liver failure

Resource category	Qty per year
**Hospital admission**	2.4^
**Specialist review**	
Initial visit	1
Subsequent visit	5
CNC	4
CNS	12
Prison guard	22
**Pathology**	
Liver function test	4
Creatitine	4
AFP	2
FBC	4
Clotting (INR, Prothrombin time)	4
Ultrasound	2
**Medication**	
Spironolactone 100mg, 100/pack	3
Propranolol, 160mg, 50/pack	3
Frusemide, 20mg, 50/pack	3
Rifaximin, 550mg, 56/pack	3
**Procedures**	
Diagnostic endoscopy (+ anaesthetic)	0·5*
Paracentesis	0·5*
Prison officers	1

^ on average per annum, * 1 in 2 patients need the procedures

Table S6: Healthcare resources requirement for hepatocellular carcinoma (HCC)

Resource category	Qty per year
**Hospital admission (diagnosis)**	2·4^
**Specialist review**	
Initial visit	1
Subsequent visit	9
CNC	4
CNS	12
Prison guard	26
Palliative care nurse	0·2*
Social worker	0·2*
**Pathology**	
Liver function test	4
Creatitine	4
AFP	2
FBC	4
Clotting (INR, Prothrombin time)	4
**Imaging**	

Ultrasound	2
CT (triple phase) or MRI	1
**Procedures**	
Diagnostic endoscopy (+ anaesthetic)	0·5**
Paracentesis	0·5**
TACE	4
**Medication**	
Sorafenib 200 mg tablet, 60/pack	6

^ on average per annum, * 1 in 5 patients required palliative care and social worker, ** 1 in 2 patients need the procedures

Table S7: Healthcare resources requirement in post-liver transplant

Resource category	Qty per year
**Hospital admission (diagnosis)**	0.05^
**Specialist review**	
Initial visit	1
Subsequent visit	9
CNC	4
CNS	12
Prison guard	26
Palliative care nurse	0·2*
Social worker	0·2*
**Pathology**	
Liver function test	4
Creatitine	4
AFP	2
FBC	4
Clotting (INR, Prothrombin time)	4
**Procedures**	
Diagnostic endoscopy (+ anaesthetic)	0·5**
**Medication**	
tacrolimus 500 microgram	12

^ on average 1 in 20 patients per annum, * 1 in 5 patients required palliative care and social worker, ** 1 in 2 patients need the procedures

4. Disease Progression Parameters

Table S8: Model parameters of HCV disease progression

Parameter	Distribution	Source/Note
Annual rate of progression from F0 to F1	Normal distribution with mean 0.128 and 95%CI: 0.080-0.176	[8]
Annual rate of progression from F1 to F2	Normal distribution with mean 0.059 and 95%CI 0.035-0.082	[8]
Annual rate of progression from F2 to F3	Normal distribution with mean 0.078 and 95%CI 0.056-0.100	[8]
Annual rate of progression from F3 to compensated cirrhosis	Normal distribution with mean 0.116 and 95%CI 0.070-0.161	[8]
Annual transition probability from compensated cirrhosis to decompensated cirrhosis	Beta (14.6168, 360.1732)	[9]Expected Value: 0.039
Annual transition probability from compensated cirrhosis or decompensated cirrhosis to HCC	Beta (1.9326, 136.1732)	[9]Expected Value: 0.014
Annual transition probability from decompensated cirrhosis or HCC to liver transplant	Beta (6.5256, 210.9945)	[9]Expected Value: 0.03
Annual rate of progression from liver transplant to post liver transplant	Uniform (1.0423, 2.4412)	[9]
Annual transition probability from decompensated cirrhosis to death	Beta (147.03, 983.97)	[9]Expected Value: 0.13
Annual transition probability from HCC to death	Beta (117.1033, 155.23)	[9]Expected Value: 0.43
Annual transition probability from liver transplant to death	Beta (16.2762, 61.2294)	[9]Expected Value: 0.21
Annual transition probability from post liver transplant to death	Beta (22.9017, 378.8825)	[9]Expected Value: 0.057
Relative risk for progression rate from compensated to decompensated cirrhosis following SVR	Lognormal distribution with mean 0.07 and 95%CI: 0.03-0.20	[10]
Relative risk for progression rate from compensated cirrhosis to HCC following SVR	Lognormal distribution with mean 0.23 and 95%CI: 0.16-0.35	[11]

Reference:

1. Yee J, Carson JM, Hajarizadeh B, et al. High Effectiveness of Broad Access Direct-Acting Antiviral Therapy for Hepatitis C in an Australian Real-World Cohort: The REACH-C Study. *Hepatol Commun* 2022; **6**(3): 496-512.
2. Hajarizadeh B, Grebely J, Byrne M, et al. Evaluation of hepatitis C treatment-as-prevention within Australian prisons (SToP-C): a prospective cohort study. *Lancet Gastroenterol Hepatol* 2021; **6**(7): 533-46.
3. New South Wales Industrial Relations Commission. Crown Employees (Correctional Officers, Corrective Services NSW) Award. Sydney, Australia; 2018.
4. Commonwealth of Australian Department of Health. Medicare Benefit Schedule Book. Canberra; 2018.
5. The Kirby Institute. Monitoring hepatitis C treatment uptake in Australia (Issue 11). Sydney, NSW, Australia: The Kirby Institute, UNSW Sydney, 2021.
6. Commonwealth of Australian Department of Health. Phrmaceutical Benefits Scheme. Canberra, Australia; 2018.
7. Independent Hospital Pricing Authority. National Efficient Price Determination 2019-20. Sydney, Australia, 2019.
8. Smith DJ, Combellick J, Jordan AE, Hagan H. Hepatitis C virus (HCV) disease progression in people who inject drugs (PWID): A systematic review and meta-analysis. *Int J Drug Policy* 2015; **26**(10): 911-21.
9. Shepherd J, Jones J, Hartwell D, Davidson P, Price A, Waugh N. Interferon alpha (pegylated and non-pegylated) and ribavirin for the treatment of mild chronic hepatitis C: a systematic review and economic evaluation. *Health Technol Assess* 2007; **11**(11): 1-205, iii.
10. van der Meer AJ, Veldt BJ, Feld JJ, et al. Association between sustained virological response and all-cause mortality among patients with chronic hepatitis C and advanced hepatic fibrosis. *JAMA* 2012; **308**(24): 2584-93.
11. Morgan RL, Baack B, Smith BD, Yartel A, Pitasi M, Falck-Ytter Y. Eradication of hepatitis C virus infection and the development of hepatocellular carcinoma: a meta-analysis of observational studies. *Ann Intern Med* 2013; **158**(5 Pt 1): 329-37.
